# Loop-mediated isothermal amplification linked a nanoparticles-based biosensor for detecting Epstein-Barr virus

**DOI:** 10.1007/s00253-023-12948-9

**Published:** 2024-01-11

**Authors:** Xinggui Yang, Xiaoyan Zeng, Junfei Huang, Ludi Yang, Sha Mao, Xu Chen, Yu Wang, Xiaoyu Wei, Shijun Li

**Affiliations:** 1 Guizhou Provincial Center for Disease Control and Prevention, Guiyang, 550004 Guizhou People’s Republic of China; 2 The Second Affiliated Hospital, Guizhou University of Traditional Chinese Medicine, Guiyang, 550003 Guizhou People’s Republic of China; 3 Tongren People’s Hospital, Tongren, 554399 Guizhou People’s Republic of China; 4 Department of Clinical Laboratory, The First People’s Hospital of Guiyang, Guiyang, 550002 Guizhou People’s Republic of China

**Keywords:** Epstein-Barr virus, Loop-mediated isothermal amplification, Gold nanoparticles–based lateral flow biosensors, EBV-LAMAD, Diagnosis

## Abstract

**Abstract:**

Epstein-Barr virus (EBV) is a ubiquitous gamma herpesvirus that maintains a lifelong latent association with B lymphocytes. Here, a rapid and reliable diagnosis platform for detecting EBV infection, employing loop-mediated isothermal amplification (LAMP) combined with a gold nanoparticles–based lateral flow biosensors (AuNPs-LFB) (termed LAMP Amplification Mediated AuNPs-LFB Detection, LAMAD), was developed in the current study. A set of specific LAMP primers targeting the Epstein-Barr nuclear antigen (EBNA) leader protein (*EBNA-LP*) gene was designed and synthesized. Subsequently, these templates extracted from various pathogens and whole blood samples were used to optimize and evaluate the EBV-LAMAD assay. As a result, the limit of detection (LoD) of the EBV-LAMAD assay was 45 copies/reaction. The EBV-LAMAD assay can detect all representative EBV pathogens used in the study, and of note, no cross-reactions were observed with other non-EBV organisms. Moreover, the whole workflow of the EBV-LAMAD assay can be completed within 70 min, including rapid EBV template preparation, EBV-LAMP amplification, and AuNPs-LFB-mediated detection. Taken together, the EBV-LAMAD assay targeting the *EBNA-LP* gene is a rapid, simplified, sensitive, reliable, and easy-to-use detection protocol that can be used as a competitive potential diagnostic/screening tool for EBV infection in clinical settings, especially in basic laboratories in resource-limited regions.

**Key points:**

• *A novel, simplified, and easy-to-use AuNPs-LFB biosensor was designed and prepared.*

• *LAMP combined with an AuNPs-LFB targeting the novel EBNA-LP gene was established.*

• *EBV-LAMAD is a rapid, sensitive, and reliable detection protocol for EBV infection.*

**Graphical Abstract:**

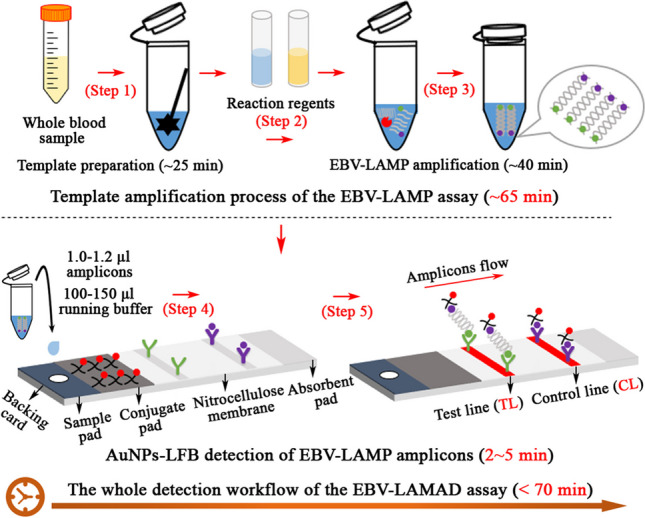

## Introduction

Epstein-Barr virus (EBV) is a ubiquitous gamma herpesvirus belonging to the genus Lymphocryptovirus, which maintains a lifelong latent association with B lymphocytes (Kimura et al. 2008; Liu et al. 2013). EBV infects over 95% of people worldwide, with greater than 90% displaying a serologic response, and its exposure typically occurs early in life (Liu et al. 2013; Nowalk and Green 2016). Usually, EBV mainly infects the pharynx by contact with respiratory secretions and then spreads throughout the body, with B lymphocytes as the primary target (Nowalk and Green 2016). Typically, EBV-infected populations are mostly asymptomatic in primary infections, occasionally leading to infectious mononucleosis (IM) in young adults or adolescents (Kimura et al. 2008; Liu et al. 2013). Of note, latent EBV infection is closely associated with the pathogenesis of many different human malignancies, such as classical Hodgkin’s lymphoma (CHL), Burkitt’s lymphoma (BL), and posttransplant lymphoproliferative disorder (PTLD), nasopharyngeal cancer (NPC), and stomach cancer, possibly because it involves in vitro and in vivo the transformation of host cells (Khanna et al. 1995b; Liu et al. 2013; Damania et al. 2022). Therefore, timely and early detection of EBV pathogens is helpful in diagnosing IM, chronic active EBV infection (CAEBV), and EBV-associated diseases.

Traditionally, the diagnosis of EBV infection has relied on clinical manifestations and serological tests; however, these protocols mentioned above inevitably suffer from time-consuming, low sensitivity, and poor reliability (frequent false-positives) (Liu et al. 2013). In particular, the diagnosis assays based on serological tests are challenging to achieve the goal of early diagnosis due to the requirement for convalescent-phase serum (Liu et al. 2013). Hence, developing a rapid, sensitive, and reliable detection technique for detecting EBV infection in populations to achieve early diagnosis is urgently needed in clinical detection settings. Currently, polymerase chain reaction (PCR) and PCR-based assays (e.g., real-time PCR and digital PCR), as conventional molecular assays, are widely used in clinical applications to detect EBV infection (Shuto et al. 2019; Yao et al. 2022). Real-time PCR assay is even utilized as the conventional method for detecting EBV infection in molecular assays (Häusler et al. 2003; Yao et al. 2022). The real-time PCR assay is relatively fast, sensitive, and specific compared to serological tests; still, the requirements for real-time PCR thermocyclers lead to it not being easy to popularize and apply in basic laboratories in resource-poor regions. Taken together, an ideal detection protocol requires fast, sensitive, and reliable characteristics, importantly also a simplified device to complete the detection workflow.

At present, the isothermal amplification techniques are able to meet these requirements as described above, like loop-mediated isothermal amplification (LAMP) (Notomi et al. 2000) and recombinase polymerase amplification (RPA) (Yang et al. 2023b). As a highly competitive diagnostic strategy, the LAMP technique has been applied to detect various pathogens like *Mycobacterium tuberculosis* (Yang et al. 2021a), SARS-CoV-2 (Huang et al. 2020), and *Candida albicans* (Fallahi et al. 2020), since it was developed by Notomi et al. in 2000. Compared to the RPA technique, the LAMP assay requires only a single reactive enzyme, resulting in a simpler amplification principle and lower detection cost (Lobato and O’Sullivan 2018; Yang et al. 2021b). In the LAMP reaction system, six primers, including forward inner primer (FIP), backward inner primer (BIP), forward outer primer (F3), backward outer primer (B3), loop F (LF), and loop B (LB), that can specifically identify eight regions of the target sequence ensure the reliability of the detection results (Notomi et al. 2000; Wang et al. 2018). However, conventional LAMP amplicon validation methods, such as real-time turbidimeter and visualization reagents (e.g., hydroxy naphthol blue, SYBR Green, and malachite-green (MG)), are difficult to accurately recognize specific and non-specific amplification due to their single working principle (Wang et al. 2017; Yang et al. 2021b). In addition, agar-gel electrophoresis is often used to identify LAMP amplicons, and the identification process is cumbersome (multi-steps) and time-consuming (~1.5 h), in addition to the shortcomings mentioned above (Iwata et al. 2006a). As a result, it is an urgent problem to design a simple, fast, and specific verification device to improve the efficiency of the LAMP detection.

In recent years, the development of gold nanoparticles–based lateral flow biosensors (AuNPs-LFB) has provided a novel strategy for accurately identifying LAMP amplicons (Li et al. 2019; Yang et al. 2023a). The AuNPs-LFB biosensor, as a simple, convenient, intuitive, reliable, and readily available verification protocol, has been widely devised to detect many pathogenic organisms in combination with nucleic acid amplification techniques like the LAMP and RPA (Wang et al. 2020; Yang et al. 2021b). In previous studies, LAMP amplification combined with an AuNPs-LFB biosensor for detecting *Brucella* spp. demonstrated high specificity due to it utilizing the reaction principle of specific binding of antigen-antibody (i.e., 6-carboxyfluorescein (FAM)/target/biotin-AuNPs complexes presented a red line after being captured by embedded anti-FITC) (Li et al. 2019; Yang et al. 2021b). Based on previous studies, the LAMP amplification combined with an AuNPs-LFB biosensor is an attractive protocol for detecting EBV infection in clinical settings.

Hence, a rapid and reliable diagnosis platform for detecting EBV infection, employing LAMP amplification combined with AuNPs-based LFB biosensors (termed LAMP, Amplification, Mediated, AuNPs-LFB, Detection, LAMAD), was developed in the current study (EBV-LAMAD). Multiple sets of specific LAMP primers targeting the novel Epstein-Barr nuclear antigen (EBNA) leader protein (*EBNA-LP*) gene were designed and synthesized. Subsequently, templates extracted from pathogens and whole blood samples and synthesized EBNA-plasmids were used to optimize and evaluate the detection performance of the LAMAD assay for EBV infection.

## Materials and methods

### Materials and instruments

Universal DNA/RNA thermostatic amplification kits and MG chromogenic reagents were purchased from Tian-Jin Huidexin Technology Development Co., Ltd. (Tianjin, China). Universal viral qEx-DNA/RNA extraction and bacterial genomic DNA extraction kits were obtained from Xi’an Tianlong Science &Technology Co., Ltd. (Xi’an, China). Commercial Epstein-Barr virus real-time PCR nuclear acid amplification kits (including nucleic acid extraction reagents) were purchased from Daan Gene Co., Ltd. (Guangzhou, China). The materials for constructing AuNPs-LFB biosensors, including a backing card, sample pad, conjugate pad, NC membrane (nitrocellulose membrane), and absorbent pad, were obtained from Jie-Yi Biotechnology. Co., Ltd. (Shanghai, China). Biotinylated bovine serum albumin (biotin-BSA) and rabbit anti-Fluorescein isothiocyanate antibody (anti-FITC antibody) were purchased from Abcam. Co., Ltd. (Shanghai, China). Dye (crimson red) streptavidin-coated gold nanoparticles (SA-AuNPs) were obtained from Bangs Laboratories, Inc. (Indiana, USA). Real-time turbidimeter (*LA*-500) was purchased by Eiken Chemical Co., Ltd. (Japan). The ChemiDoc MP imaging system was provided by Bio-Rad (USA).

### Design and assembly of the AuNPs-based LFB biosensor

The AuNPs-based LFB biosensor was designed and assembled according to the specific binding principle of antigens (FITC/FAM and biotin) to antibodies (anti-FITC antibodies and biotin-BSA) (Wang et al. 2018; Yang et al. 2022). Briefly, these biomaterials were fixed on the backing card using a solid adhesive, including the sample pad, conjugate pad embedded with SA-AuNPs polymers (129 nm, 10 mg mL^−1^, 100 mM borate, pH 8.5 with 0.1% BSA, 0.05% Tween 20, and 10 mM EDTA), NC membrane, and absorbent pad. There are two reaction regions with a spacing of 5 mm on the NC membrane, including a test region embedded with anti-FITC antibiotics (2.5 mg/ml) to form a test line (TL) and a control region embedded with biotin-BSA (0.15 mg/ml) to form a control line (CL). According to the design mentioned above, the AuNPs-LFB biosensors were assembled by TianJin HuiDeXin Biotech. Co., Ltd. (Tianjin, China), and the assembled LFB biosensor were stored in a dry environment (4–30 °C) for use within 1 year.

### Design of the LAMP primers and EBNA-plasmids

Multiple sets of EBV-LAMAD primers targeting the *EBNA-LP* gene (GenBank accession number, MK973061.1, 21238-24310), including FIP* (F1c binding to F2), BIP (B1c binding to B2), F3, B3, LF*, and LB primers, were designed using the LAMP primer online design tools (http://primerexplorer.jp/lampv5e/index.html). The sequence alignment of designed EBV-LAMAD primers was performed using the BLAST software (basic local alignment search tool). Then, five sets of EBV-LAMAD primers were synthesized for screening the optimal EBV-LAMAD primers used in the current study. According to the design principle of AuNPs-LFB biosensor, the 5′ end of FIP* primers is labeled with FAM groups, and the 5' end of LF* primers is labeled with biotin in the optimal set of EBV-LAMAD primers. The detailed information on optimal EBV-LAMAD primers, including name, sequence, length, and modification sites, is presented in Table 1.. The EBV-LAMAD primers (HPLC purification grade) were synthesized by Tiany-Huiyuan Biotech Co., Ltd. (Beijing, China). Moreover, the EBNA-plasmids were prepared using a partial sequence of the *EBNA-LP* gene (GenBank accession number, MK973061.1) that covers the regions recognized by all EBV-LAMAD primers. The preparation process, including amplification, enzyme digestion, recombination (pUC57), transformation, and EBNA-plasmids extraction, was performed by Tianyi-Huiyuan Biotech Co., Ltd. (Beijing, China). The original plasmids obtained were diluted and quantified to prepare a series of diluents (3×10^8^, 3×10^7^, 3×10^6^, 3×10^5^, 3×10^4^, 3×10^3^, 3×10^2^, 3×10^1^, 3×10^0^, and 3×10^−1^ copies) using Tris-EDTA buffer (1×).

**Table 1:** Primers used in this study

Primers ^a^	Sequences and modifications (5′- 3′)	Length ^b^
EBNA-F3	AGGAATAAGCCCCCAGACA	19 nt
EBNA-B3	ACCAGAAATAGCTGCAGGAC	20 nt
EBNA-FIP*	FAM-TAGCAACGCGAACCCCCTTGGGGGAGTGGGCTTGTTTG	38 mer
EBNA-BIP	CTCAGTCCAGCGCGTTTACGTCTTTATACCAGGGGCAGTGG	41 mer
EBNA-LF*	Biotin-CCCTGACCTTTGGTGAAGTCA	21 nt
EBNA-LB	AAGCCAGACAGCAGCCA	17 nt

^a^FAM, 6-Carboxyfluorescein

^b^*nt*, nucleotide; *mer*, monomeric unit

### Extraction of nucleic acid templates

Twenty-one organisms were used here to evaluate the specificity of the EBV-LAMAD assay (Table 2). The nucleic acid templates of these pathogens were extracted using the viral qEx-DNA/RNA extraction and universal bacterial genomic DNA extraction kits according to the operation directions. In addition, a total of 101 whole blood samples were used to evaluate the capability of the EBV-LAMAD assay for detecting EBV infection in clinical applications. These DNA templates of whole blood samples were prepared using commercial Epstein-Barr virus nucleic acid extraction reagents (Daan Gene Co., Ltd. (Guangzhou, China). Simply put, 2 ml of whole blood sample diluted with 1 ml of 0.9% NaCl solution was slowly added to a glass test tube containing 500 μl of lymphocyte separation medium (Tianjin Haoyang Biological Manufacture Co., Ltd. (Tianjin, China)) and centrifuged at 2000 rpm for 10 min. Then, the leukocyte-rich solution (second layer below the liquid surface) was pipetted into a 1.5-ml centrifuge tube and centrifuged at 12,000 rpm for 5 min; then, the supernatant was discarded, 50 μl of DNA extraction solution was added and mixed, and heated at 100 °C for 8 min. Finally, the heated test tube was centrifuged at 12,000 rpm for 2 min, and the supernatant was stored at −20 °C until use before.

**Table 2:** Pathogens used in this study

Pathogens	Source of strains ^a^	No. of strains
Target pathogens		
Epstein-Barr virus	Standard culture (BioBDS)	1
Epstein-Barr virus	CHCIP	1
Epstein-Barr virus	Positive samples	4
Non-target pathogens		1
Human cytomegalovirus	BNCC	1
Parainfluenza virus 1	CHCIP	1
Parainfluenza virus 3	CHCIP	1
Influenza B virus	CHCIP	1
Rubella virus	CHCIP	1
Coxsackievirus A16	CHCIP	1
Sendai virus	CHCIP	1
Respiratory syncytial virus	CHCIP	1
*Pseudomonas aeruginosa*	GZCDC	1
*Streptococcus pneumoniae*	GZCDC	1
*Klebsiella Pneumoniae*	GZCDC	1
*Mycobacterium tuberculosis*	GZCDC	1
*Staphylococcus aureus*	GZCDC	1
*Brucella melitensis*	GZCDC	1
*Salmonellae* spp.	GZCDC	1
Total		21

^a^*BioBDS*, BioBDS Biotechnology Co., Ltd. (Guangzhou, China); *CHCIP*, Children’s Hospital Capital Institute of Pediatrics; *BNCC*, BeNa Culture Collection; *GZCDC*, Guizhou Provincial Center for Disease Control and Prevention

### The EBV-LAMAD reaction

The 25-μl amplification mixture of the EBV-LAMAD assay contains the following: 12.5 μl of 2 × amplification buffer, 1 μl of *Bst* thermostatic enzyme (2.0), 2.0 μM each of FIP* and BIP, 1.0 μM each of LF* and LB, 0.5 μM each of F3 and B3, 1.5 μl of nucleic acid templates extracted from pure culture, and 5 μl of nucleic acid templates extracted from whole blood samples, and double-distilled water was added to 25 μl. The mixed system of EBV-LAMAD assay was amplified at 63 °C for 60 min. Then, the EBV-LAMAD amplicons were validated using an AuNPs-LFB biosensor, MG chromogenic regents, 1.5% agar-gel electrophoresis, and real-time turbidimeter (*LA-*500).

### Confirmation experiment of the EBV-LAMAD assay

To confirm the feasibility of the designed EBV-LAMAD assay, the validation test was carried out according to the EBV-LAMAD reaction system. Here, a total of four different reactions were implemented simultaneously, including positive reaction (1.5 μl of EBNA-plasmids), negative control (1.5 μl of templates extracted from Human cytomegalovirus), laboratory internal control (1.5 μl of environmental samples in this laboratory), and blank control (1.5 μl of double-distilled water). The reaction tubes of the EBV-LAMAD assay were incubated at 63 °C for 60 min. Finally, these results of the EBV-LAMAD assay were reported using an AuNPs-LFB biosensor, MG chromogenic regents, 1.5% agar-gel electrophoresis, and a real-time turbidimeter (*LA-*500), respectively.

### Optimization test of the EBV-LAMAD assay

Firstly, to obtain the best amplification temperature, the EBV-LAMAD assay was optimized based on the LAMP reaction system. The EBV-LAMAD assay was performed at nine different incubation temperatures (62–70 °C at 1 °C intervals), respectively. Here, 1.5 μl of EBNA-plasmids (3×10^5^ copies) was used as the amplification template, and the reaction results were monitored by implementing a real-time turbidimeter (*LA-*500). Moreover, the optimization tests of time for the EBV-LAMAD assay were also performed by setting different incubation times (10–60 min with intervals of 10 min). Similarly, 1.5 μl diluents of each EBNA-plasmids (3×10^8^, 3×10^7^, 3×10^6^, 3×10^5^, 3×10^4^, 3×10^3^, 3×10^2^, 3×10^1^, 3×10^0^, and 3×10^−1^ copies) were used as amplification templates for the EBV-LAMAD assay. The amplification results of the EBV-LAMAD assay were reported using the AuNPs-LFB biosensor.

### Sensitivity test of the EBV-LAMAD assay

In the current study, the detection sensitivity of the EBV-LAMAD assay was tested according to the optimal reaction temperature and time (i.e., amplification at 66 °C for 40 min). A series diluent of 1.5 μl of EBNA-plasmids (3×10^8^, 3×10^7^, 3×10^6^, 3×10^5^, 3×10^4^, 3×10^3^, 3×10^2^, 3×10^1^, 3×10^0^, and 3×10^−1^ copies) was used as the amplification template. Finally, the amplification results were validated using an AuNPs-LFB biosensor, MG chromogenic regents, 1.5% agar-gel electrophoresis, and a real-time turbidimeter (*LA-*500). Parallel experiments were repeated more than three times.

### Specificity test of the EBV-LAMAD assay

A total of twenty-one organisms (including Epstein-Barr virus and other non-EBV) were used to evaluate the specificity of the EBV-LAMAD assay. Specificity tests were performed according to the EBV-LAMAD reaction system and the optimal reaction conditions. Subsequently, the reaction results were tested using AuNPs-LFB biosensors.

*Applicability of the EBV-LAMAD assay for detecting EBV infection in whole blood samples*

Here, 101 whole blood samples with suspected EBV infection were used to evaluate the practical capability of the EBV-LAMAD assay (Table 3). According to the extraction steps described above, 5 μl nucleic acid templates extracted from these samples were used as amplification templates for EBV-LAMAD and real-time PCR assays. The EBV-LAMAD assay for detecting EBV infection was carried out based on the optimal reaction conditions of the LAMP assay, and the amplicons were reported using the AuNPs-LFB biosensor. Moreover, the real-time PCR assay was also used as the standard method for diagnosing EBV infection or non-EBV cases in the current study, and the detection procedure is performed according to the operating instructions (Daan Gene Co., Ltd. (Guangzhou, China)). Finally, the detection sensitivity and specificity of the EBV-LAMAD assay were evaluated by comparing the results of the real-time PCR assay.

**Table 3:** Evaluation of the applicability of the EBV-LAMAD assay for testing whole blood samples

Methods ^a^	Real-time PCR assay ^b^		Sensitivity (%)	Specificity (%)
	EBV cases (*n*=26)	non-EBV cases (*n*=75)		
EBV-LAMAD assay				
Positive	26	0	100	100
Negative	0	75	100	100

^a^*LAMAD*, LAMP, Amplification, Mediated, AuNPs-LFB, Detection; *LAMP*, loop-mediated isothermal amplification; *AuNPs-LFB*, gold nanoparticles–based lateral flow biosensor

^b^*PCR*, polymerase chain reaction

## Results

### Overview of reaction mechanism of EBV-LAMAD assay

**Fig. 1:**
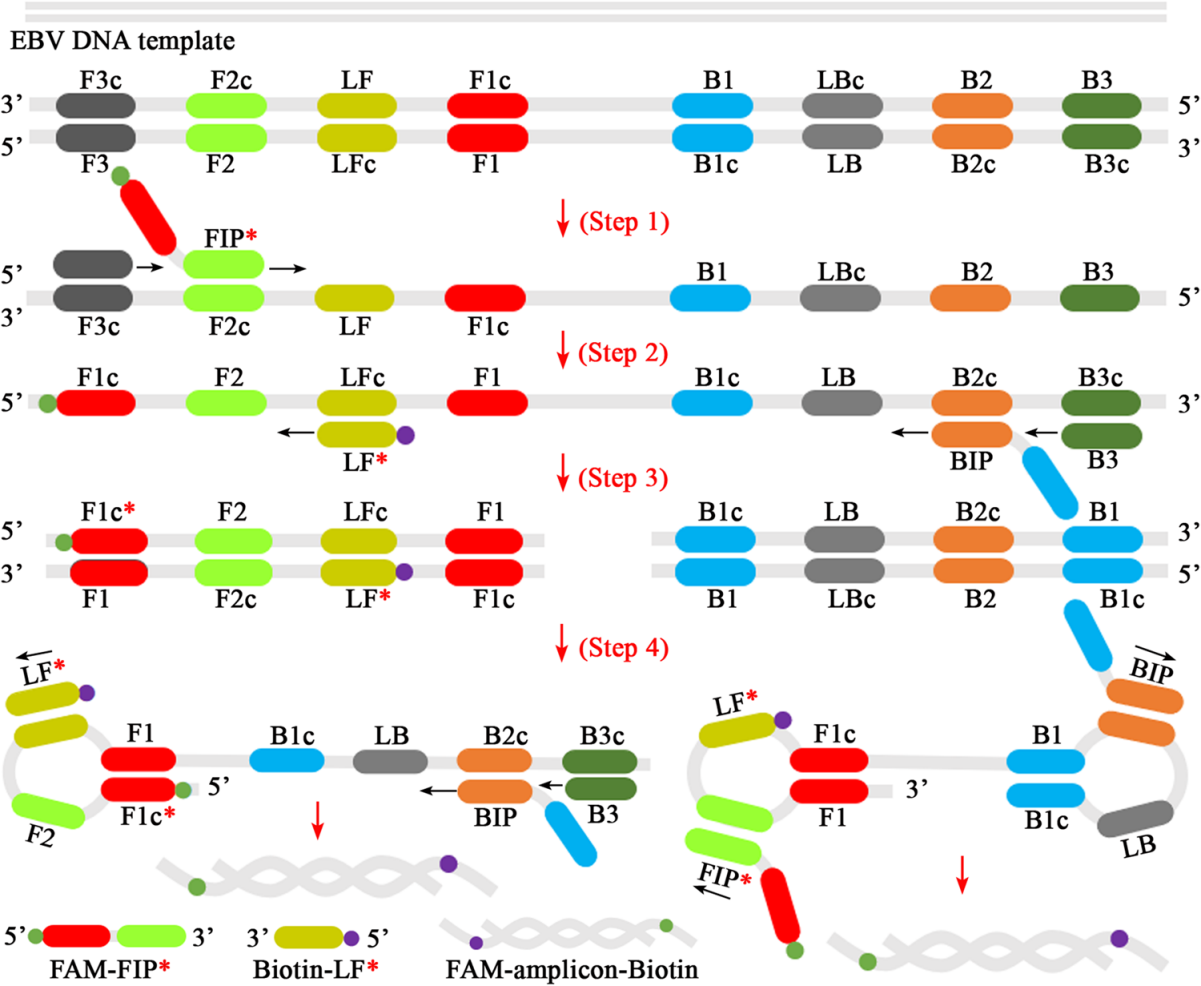
Overview of the reaction principle of the EBV-LAMAD assay. Six LAMP primers (including FIP*, BIP, LF*, LB, F3, and B3) can bind to ten complementary regions on the *EBNA-LF* gene (step 1). These LAMP primers bound to the target template were further extended to synthesize new strands under the trigger of *Bst* DNA polymerase in EBV-LAMAD reaction systems (step 2). The FIP* primers labeled with FAM can extend forward and synthesize new strands containing FAM labels (step 2). Then, the LF* primers labeled with biotin were complementary to the newly synthesized strands labeled with FAM and extended in reverse (step 3). According to the above reaction principle, the FAM-amplicons-biotin complexes that can be captured by anti-FITC antibodies were produced exponentially in the LAMP reaction system (steps 3 and 4). FAM, 6-carboxyfluorescein; LAMAD, LAMP Amplification Mediated AuNPs-LFB Detection; LAMP, loop-mediated isothermal amplification; AuNPs-LFB, gold nanoparticles–based lateral flow biosensor

**Fig. 2:**
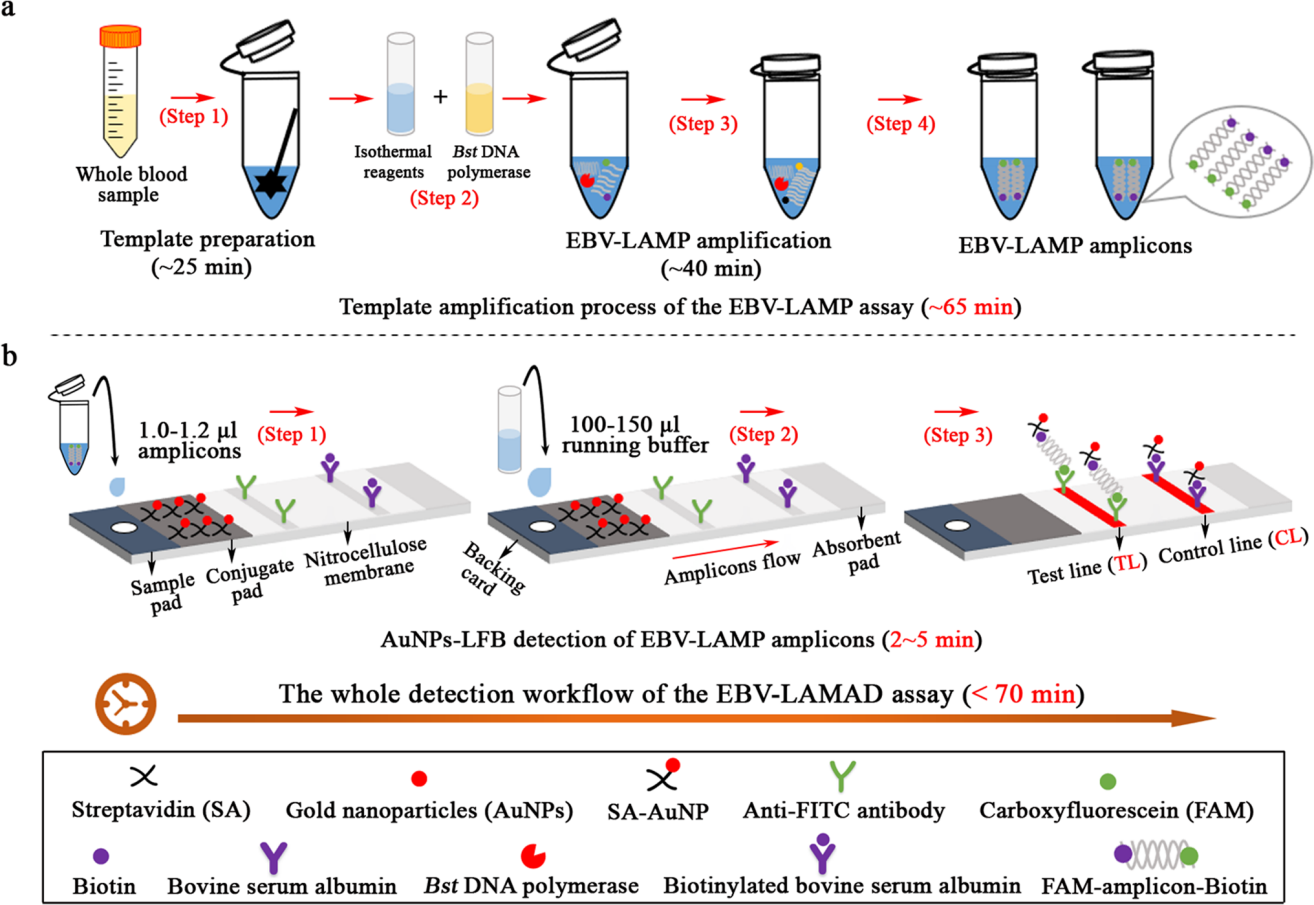
Schematic diagram of detection workflow of the EBV-LAMAD assay. The workflow for the EBV-LAMAD assay consists of two main parts; (1) the EBV-LAMAD reaction system with premixed EBV templates and isothermal reagents was incubated at 66°C for 40 min (steps 1, 2, 3, and 4); (2) validation of EBV-LAMAD amplicons mediated by AuNPs-LFB biosensor (steps 1, 2, and 3); here, two results were generated, including a positive reaction (both TL and CL lines were red) and a negative reaction (only CL line was red, while TL line is colorless) in AuNPs-LFB biosensor. LAMAD, LAMP Amplification Mediated AuNPs-LFB Detection; LAMP, loop-mediated isothermal amplification; AuNPs-LFB, gold nanoparticles–based lateral flow biosensor; TL, test line; CL, control line

In the EBV-LAMAD reaction system, the specific EBV-LAMP primers were able to recognize eight different regions of the target sequence (namely, F3c, F2c, LFc, F1c, LBc, B1c, B2c, and B3c) (Fig. 1, step 1). These LAMP primers bound to the target template were further extended to synthesize new strands under the trigger of *Bst* DNA polymerase in EBV-LAMAD reaction systems (Fig. 1, step 2). Importantly, the FIP* primers labeled with FAM can extend forward and synthesize new strands containing FAM labels (Fig. 1, step 2). Then, the LF* primers labeled with biotin were complementary to the newly synthesized strands labeled with FAM and extended in reverse (Fig. 1, step 3). According to the above reaction principle, the FAM-amplicons-biotin complexes that can be captured by anti-FITC antibodies were produced exponentially in the LAMP reaction system (Fig. 1, steps 3 and 4). In general, the detection workflow for the EBV-LAMAD assay consists of two main parts; (1) the EBV-LAMAD reaction system with premixed EBV templates and isothermal reagents was incubated at 66 °C for 40 min (Fig. 2a, steps 1, 2, 3, and 4); (2) validation of EBV-LAMAD amplicons mediated by AuNPs-LFB biosensor (Fig. 2b, steps 1, 2, and 3); here, two results were generated, including a positive reaction (both TL and CL lines were red) and a negative reaction (only CL line was red, while TL line is colorless) in AuNPs-LFB biosensor.

### Assessment test of the EBV-LAMAD assay

**Fig. 3:**
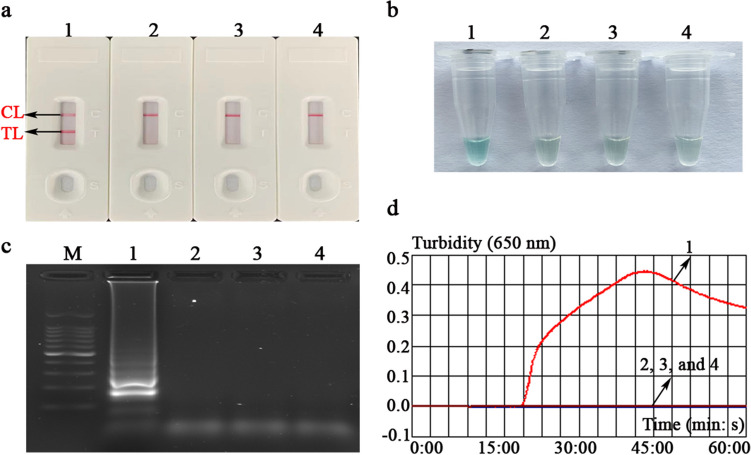
Confirmation test of the EBV-LAMAD assay. The EBV-LAMAD products were reported by AuNPs-LFB biosensor (a), MG chromogenic reagent (b), 1.5% agar-gel electrophoresis (c), and real-time turbidimeter (d). Strip (a1)/tube (b1)/lane (c1)/line (d1), positive amplification of the EBV-LAMAD assay using the EBNA*-*plasmids; strip (a2)/tube (b2)/lane (c2)/line (d2), negative control of the EBV-LAMAD assay using the nucleic acid template extracted from Human cytomegalovirus; strip (a3)/tube (b3)/lane (c3)/line (d3), negative control of the EBV-LAMAD assay using the environmental sample in the test; strip (a4)/tube (b4)/lane (c4)/line (d4), blank control of the EBV-LAMAD assay using double-distilled water. Lane **M**, 100 bp DNA ladder; LAMAD, LAMP Amplification Mediated AuNPs-LFB Detection; LAMP, loop-mediated isothermal amplification; AuNPs-LFB, gold nanoparticles–based lateral flow biosensor; TL, test line; CL, control line

In evaluation tests of the EBV-LAMAD assay, the AuNPs-LFB biosensors, MG chromogenic regents, 1.5% agar-gel electrophoresis, and real-time turbidimeter (*LA-*500) were used to validate EBV-LAMP amplicons, respectively (Fig. 3). Here, the verification results of four methods are consistent. In the AuNPs-LFB biosensor, two red lines (TL and CL lines) appeared in a positive reaction, and only CL line showed red in the negative, internal, and blank controls (Fig. 3a). In the MG chromogenic reagent system, positive amplification is blue, and negative amplification is colorless or light blue (Fig. 3b). Meanwhile, the characteristic gradient lane can be observed in positive reactions when using 1.5% agar-gel electrophoresis to validate the EBV-LAMP amplicons (Fig. 3c). Moreover, the turbidity value of positive reaction was greater than 0.1; conversely, the turbidity was less than 0.1 in the negative, internal, and blank controls (Fig. 3d).

### Optimal reaction conditions of the EBV-LAMAD assay

**Fig. 4:**
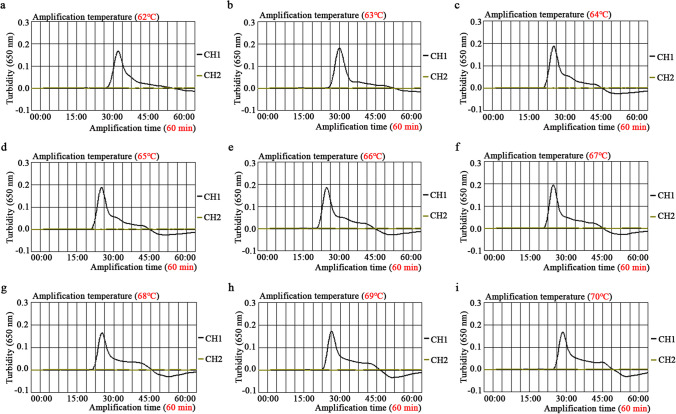
Temperature optimization of the EBV-LAMAD assay. Here, nine dynamic curves (a–i) were generated using different amplification temperatures (62–70 °C, 1 °C interval). A turbidity value greater than 0.1 was considered positive amplification, while a turbidity value less than 0.1 was regarded as a negative reaction in the current study. The detection efficiency of the EBV-LAMAD assay was better than other temperatures when the temperature was 66°C (e). LAMAD, LAMP Amplification Mediated AuNPs-LFB Detection; LAMP, loop-mediated isothermal amplification; AuNPs-LFB, gold nanoparticles–based lateral flow biosensor; TL, test line; CL, control line

**Fig. 5:**
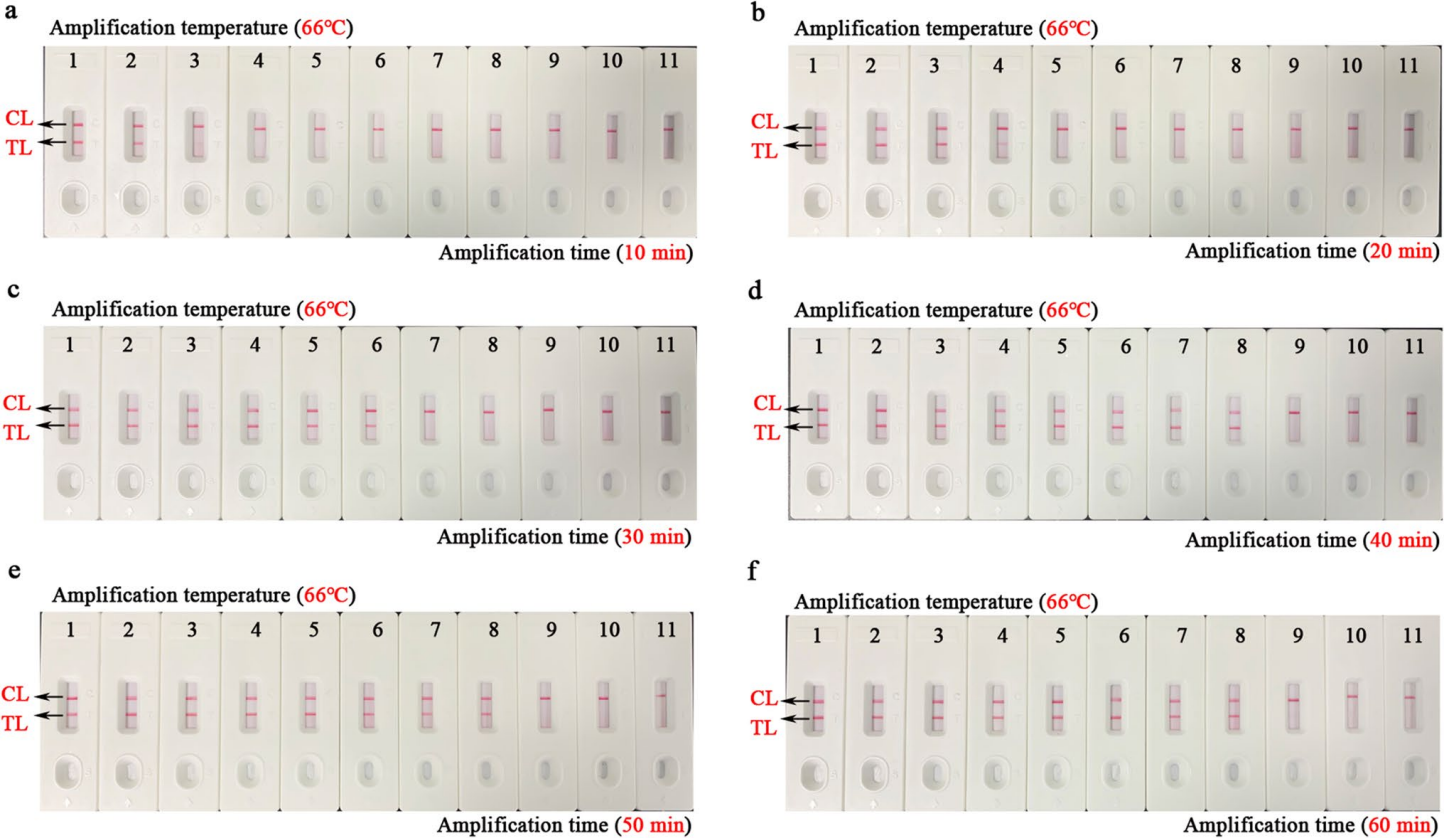
Time optimization of the EBV-LAMAD assay. The reaction time of EBV-LAMAD assay was optimized by detecting EBNA*-*plasmids diluents (namely, 3×10^8^, 3×10^7^, 3×10^6^, 3×10^5^, 3×10^4^, 3×10^3^, 3×10^2^, 3×10^1^, 3×10^0^, and 3×10^−1^ copies). The plots (a–f) correspond to different reaction times (10–60 min, 10-min intervals). Strips 1–10 (a–f) correspond to 3×10^8^, 3×10^7^, 3×10^6^, 3×10^5^, 3×10^4^, 3×10^3^, 3×10^2^, 3×10^1^, 3×10^0^, and 3×10^−1^ copies. Strips 11 (a–f) correspond to double-distilled water. The lowest concentration of EBNA-plasmids that can be detected by EBV-LAMAD assay is 45 copies/reaction at a time ranging from 40 to 60 min. As a result, the optimal reaction time of the EBV-LAMAD assay was 40 min in the study. LAMAD, LAMP Amplification Mediated AuNPs-LFB Detection; LAMP, loop-mediated isothermal amplification; AuNPs-LFB, gold nanoparticles–based lateral flow biosensor; TL, test line; CL, control line

As shown in Fig. 4, when the temperature was 6 6°C, the amplification efficiency of the EBV-LAMAD assay is optimal in the nine dynamic curves generated in the current study. In addition, a total of six plates of reaction time were generated by AuNPs-LFB biosensors in the time optimization test. When the amplification time was 40 min, the lowest concentration of the diluents of EBNA-plasmids detected by EBV-LAMAD assay was 45 copies/reaction, consistent with the amplification time of 50 and 60 min (Fig. 5). As a result, the optimal reaction condition for the EBV-LAMAD assay was 66 °C for 40 min.

### Sensitivity and specificity of the EBV-LAMAD assay

**Fig. 6:**
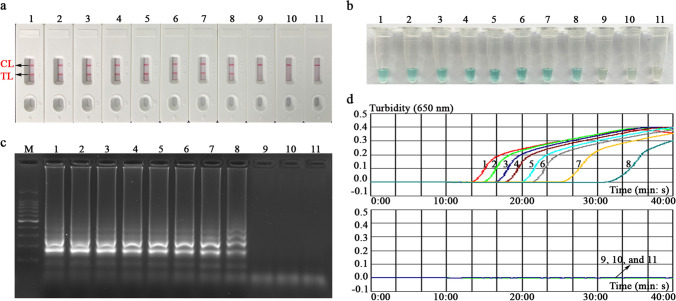
Analytical sensitivity of the EBV-LAMAD assay. The sensitivity of EBV-LAMAD assay was tested using different concentrations of EBNA*-*plasmids, and the amplicons were validated using an AuNPs-LFB biosensor (a), MG chromogenic reagent (b), 1.5% agarose gel electrophoresis (c), and a real-time turbidimeter (d). Strips (a1–a10)/tubes (b1–b10)/lanes (c1–c10)/lines (d1–d10) correspond to 3×10^8^, 3×10^7^, 3×10^6^, 3×10^5^, 3×10^4^, 3×10^3^, 3×10^2^, 3×10^1^, 3×10^0^, and 3×10^−1^. Strips (a11)/tubes (b11)/lanes (c11)/lines (d11) correspond to nuclease-free water. **Lane M**, 100 bp DNA ladder; LAMAD, LAMP Amplification Mediated AuNPs-LFB Detection; LAMP, loop-mediated isothermal amplification; AuNPs-LFB, gold nanoparticles–based lateral flow biosensor; TL, test line; CL, control line

**Fig. 7:**
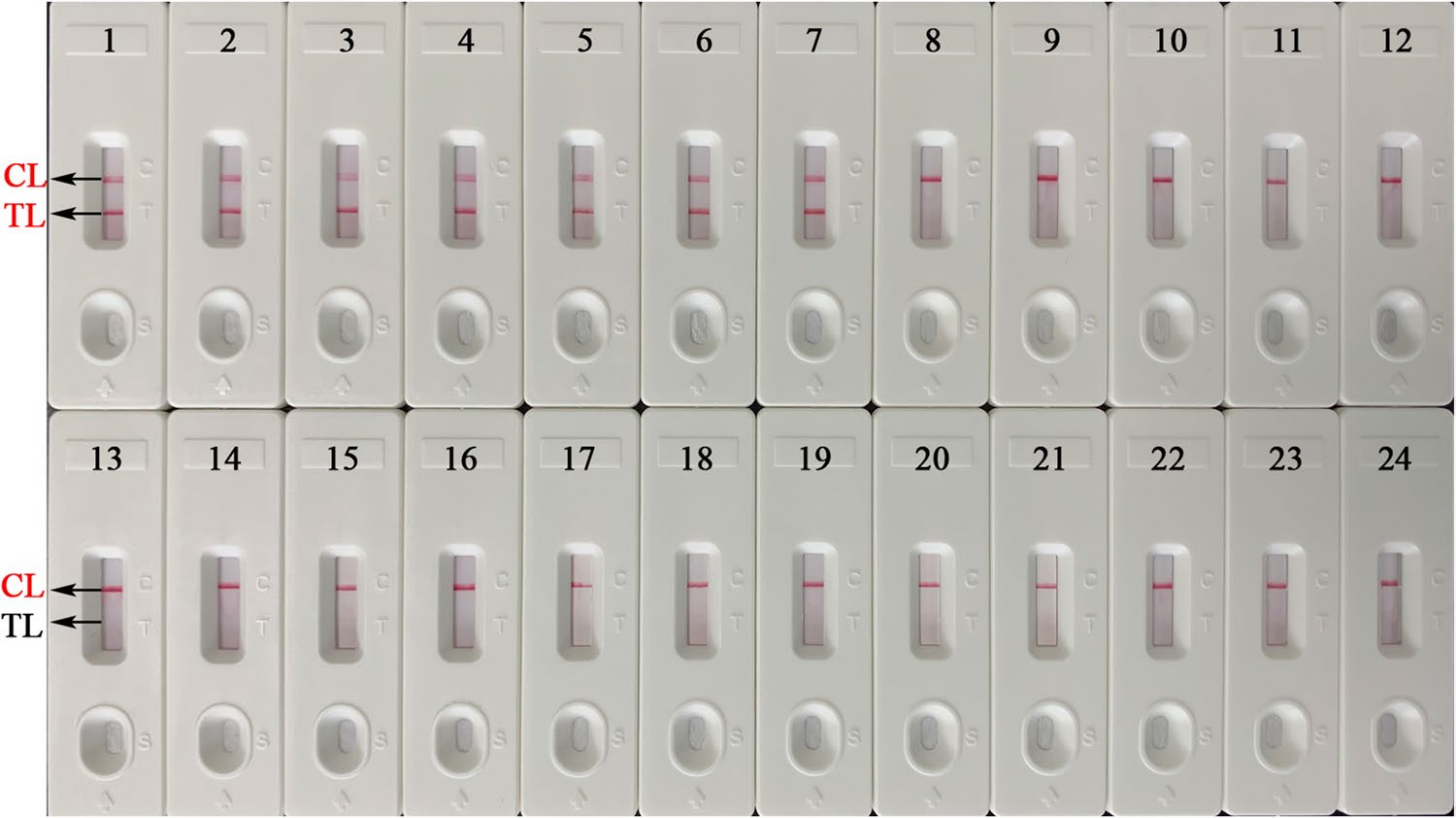
Analytical sensitivity of the EBV-LAMAD assay. The specificity of EBV-LAMAD assay was tested using 21 pathogens, and the LAMP products were reported using AuNPs-LFB biosensors. Strip 1, EBV (inactivated standard EBV culture); strip 2, EBV template (CHCIP); strip 3, EBNA*-*plasmids, strips 4–7, EBV-positive clinical samples; strips 8–15, Human cytomegalovirus, Parainfluenza virus 1, Parainfluenza virus 3, Influenza B virus, Rubella virus, Coxsackievirus A16, Sendai virus, Respiratory syncytial virus; strips 16–22, *Pseudomonas aeruginosa*, *Streptococcus pneumoniae*, *Klebsiella Pneumoniae*, *Mycobacterium tuberculosis*, *Staphylococcus aureus*, *Brucella melitensis*, *Salmonellae* spp.; strips 23 and 24, negative control (environmental sample in the test) and blank control (double-distilled water). LAMAD, LAMP Amplification Mediated AuNPs-LFB Detection; LAMP, loop-mediated isothermal amplification; AuNPs-LFB, gold nanoparticles–based lateral flow biosensor; TL, test line; CL, control line; GZCDC, Guizhou Provincial Center for Disease Control and Prevention; CHCIP, Children’s Hospital Capital Institute of Pediatrics

To explore the capability of the EBV-LAMAD assay to detect templates at lower concentrations, this sensitivity test was performed in the current study. In sensitivity tests, the detection of limit (LoD) of the EBV-LAMAD assay was 45 copies/reaction (Fig. 6). Furthermore, as shown in Fig. 7, the EBV-LAMAD assay can detect inactivated EBV standard culture (BioBDS), EBV (CHCIP), EBNA-plasmids, and EBV-positive clinical samples (Table 2). Of note, no cross-amplifications were observed with other non-EBV pathogens, including closely related Human cytomegalovirus. These data confirmed that the detection specificity of the EBV-LAMAD assay was 100%.

### Evaluation of the EBV-LAMAD assay for detecting whole blood samples

In the current study, a total of 101 nucleic acid templates extracted from whole blood samples with suspected EBV infection were examined by EBV-LAMAD and real-time PCR assays. Here, twenty-six whole blood samples were detected as EBV-positive, while 75 were EBV-negative by real-time PCR assay. These 26 whole blood samples diagnosed as positive by real-time PCR were determined as EBV infection patients. In comparison, the remaining 75 PCR-negative samples were included in non-EBV control cases. Similarly, the 26 samples collected from EBV infection patients were also tested as EBV-positive and 75 specimens collected from non-EBV cases were EBV-negative by EBV-LAMAD assay (Table 3). The detection sensitivity and specificity of the EBV-LAMAD assay were 100%. Notably, whole blood samples with low viral load (approximately 500 to 1000 copies) quantified by real-time PCR were also tested as positive by EBV-LAMAD assay. In summary, these data confirmed the reliability of the EBV-LAMAD assay developed in the study for detecting EBV infection in whole blood samples.

## Discussion

Currently, EBV infection cannot be ignored in the population, as its latent infection is closely related to many different human malignancies like CHL, BL, PTLD, and NPC (Khanna et al. 1995a; Kimura et al. 2008; Liu et al. 2013). As a result, early, rapid, sensitive, specific diagnosis of EBV infection has a critical role in diagnosing IM-, CAEBV-, and EBV-related diseases. The LAMP technique, as an attractive detection protocol, has been widely designed and applied to detect various pathogens in previous studies (Li et al. 2019; Fallahi et al. 2020; Huang et al. 2020; Yang et al. 2023a). This technique showed high sensitivity and specificity due to its ability to recognize multiple regions on the target sequence and simultaneously perform multidirectional amplification triggered by *Bst* DNA polymerase (Notomi et al. 2000; Wang et al. 2018). In early studies, Iwata et al. combined conventional LAMP technology with agar-gel electrophoresis to detect EBV infection, demonstrating preferable detection capabilities in clinical applications (Iwata et al. 2006a). Usually, validation methods for LAMP amplicons similar to agar-gel electrophoresis, such as visualization reagents and real-time turbidity, are applied as conventional protocols (Iwata et al. 2006a; Liu et al. 2013). However, due to the single action mechanism of the above validation strategies, it is not easy to accurately distinguish between LAMP-specific and non-specific amplification (Wang et al. 2017; Li et al. 2019). Hence, developing a novel and accurate verification method is an urgent problem for the LAMP assay.

In the current report, the AuNPs-based LFB biosensor designed can overcome the shortcomings of the above approaches because its embedded anti-FITC can specifically bind to the FAM/amplicons/biotin complexes (Fig. 2b). Of note, the verification of AuNPs-LFB biosensors for EBV-LAMP amplicons was highly consistent with the MG chromogenic reagent, real-time turbidity, and agar-gel electrophoresis used in this study, confirming its reliability in practical application (Fig. 3). Although real-time turbidity can monitor the amplification process of EBV-LAMAD assay, it is challenging to meet the detection needs of basic laboratories in resource-poor regions due to its reliance on specialized real-time turbidimeters. Compared with agar-gel electrophoresis, the validation strategy of AuNPs-LFB biosensor significantly shortens the detection time (2–5 min) and simplified the detection procedures (Fig. 2b). Moreover, the internal control (i.e., CL line, embedded biotin-BSA bind to biotin from the conjugate pad) was introduced into AuNPs-LFB biosensor to ensure the validity of the test results (Fig. 2b). Nevertheless, open-tube detection may be associated with carryover contamination when the AuNPs-LFB biosensor is used to validate EBV-LAMAD amplification products (Yang et al. 2021b). Thus, the laboratory zones need to be strictly distinguished to reduce the possibility of cross-reactions caused by carryover contamination. In the current study, the experiment zones of the EBV-LAMAD assay were mainly divided into sample preparation, reaction system premixing, EBV-LAMP amplification, and product validation zones. Laboratory zoning is also a reliable, simplified, and economical physical strategy that eliminates carryover contamination from nucleic acid amplification techniques (NAATs) like PCR, PCR-based, and LAMP. So far, carryover contamination has never occurred in our conventional detection after implementing the strategy.

However, as many nucleic acid–based detection methods like PCR and PCR-based assays do, LAMP detection results are easily affected by the detection target (Zhang et al. 2015). In general, the detection accuracy is most affected mainly by the mutations of primer-covered regions in the target genes (Zhang et al. 2015). Therefore, selecting a target sequence is extremely important for all nucleic acid–based detection methods. Previously, many sequences, including *BamHI W*, *EBNA1* (*BamHI K* region), and *BALF5*, were used as detection targets of EBV pathogen (Telenti et al. 1990; Iwata et al. 2006b; Liu et al. 2013). Nevertheless, the exploration of a novel detection target and its successful combination with LAMP assay can provide different detection protocols for EBV detection in clinical applications. The *EBNA-LP* gene, consisting of W1W2 repeats and a unique C-terminal Y1Y2 domain, is one of the first viral genes expressed upon B-cell infection (Kawaguchi et al. 2000; Szymula et al. 2018). Specific LAMP primers targeting the novel *EBNA-LP* gene (GenBank accession number, MK973061.1, 21238-24310) were designed and synthesized after the target-region conservatism was analyzed using BLAST software (Table 1.). As expected, the EBV-LAMAD assay targeting the novel *EBNA-LP* gene demonstrated excellent specificity for detecting EBV pathogens in our evaluation tests (Fig. 3).

In sensitivity tests, the EBV-LAMAD assay demonstrating excellent sensitivity using the optimal reaction conditions (66 °C, 40 min) can detect the EBNA-plasmids at a minimum concentration of 45 copies/reaction (Figs. 4, 5, and 6). Compared to real-time PCR (approximately 500 copies), the EBV-LAMAD assay has a higher sensitivity, resulting in better detection capability for specimens with low viral loads. Moreover, the EBV-LAMAD assay can detect EBNA-plasmids and all nucleic acid templates extracted from EBV pathogens used in the study (Fig. 7 and Table 2). Importantly, no cross-reactions were observed with other representative non-EBV-pathogens used in the study, thus demonstrating its high specificity (Fig. 7 and Table 2). Overall, these test data confirmed that the EBV-LAMAD assay established in this study is a highly sensitive and specific assay that can be used as a reliable tool for detecting EBV pathogens. Currently, an advanced EBV-LAMP detection system that avoids nucleic acid extraction has been developed to diagnose Endemic Burkitt Lymphoma (eBL), termed cell-to-LAMP assay (Gardner et al. 2022). Still, its excessive dependence on specific cells makes it challenging to detect different types of samples (e.g., nasopharyngeal swabs).

Generally, the real-time PCR assay is still employed as a conventional method for detecting many pathogens (e.g., EBV, SARS-CoV-2, influenza virus) in clinical settings due to its high reliability (Vemula et al. 2016; Li et al. 2020; Tonoyan et al. 2020). In the current study, EBV real-time PCR assay was used as a standard method to objectively evaluate the established EBV-LAMAD assay. The experimental data confirmed the EBV-LAMAD assay has high practicality for detecting EBV infection in whole blood samples (detection specificity and sensitivity were both 100%) by comparing it with the results of the real-time PCR assay (Table 3). Although EBV-LAMAD exhibits excellent detection sensitivity and specificity for whole blood samples in the current study, it is limited by the sample size. In the future, if the EBV-LAMAD technique is further applied in clinical settings, more samples should be collected to validate its sensitivity and specificity further if conditions permit. Admittedly, the inability to achieve quantitative detection is also a drawback of EBV-LAMAD assay compared to real-time PCR test; still, due to the detection aims to diagnose EBV infection in clinical applications, thus quantifying EBV DNA is not necessary. Moreover, the entire workflow of the EBV-LAMAD assay can be completed within 70 min, including rapid EBV template preparation, EBV-LAMP amplification, and AuNPs-LFB-mediated validation for LAMP amplicons. Compared to real-time PCR assays for detecting EBV infection (~ 2 h), the EBV-LAMAD assay is less time-consuming and more accessible because it does not require specialized heating equipment (such as the necessary thermal cycler for PCR assay). The data demonstrated that the EBV-LAMAD assay is a rapid, simplified, sensitive, reliable, and easy-to-obtain detection method that can be used as a competitive potential diagnosis/screening tool for EBV infection in clinical applications.

In conclusion, a novel detection strategy called the EBV-LAMAD assay, which utilizes LAMP amplification combined with an AuNPs-LFB biosensor, was successfully established and applied to detect EBV infection in this report. In the EBV-LAMAD system, a simple, reliable, easy-to-use AuNPs-LFB biosensor was designed and applied to simplify test procedures and improve detection efficiency. Moreover, the EBV-LAMAD assay established in this study demonstrated high sensitivity and specificity for detecting EBV pathogens. Taken together, the EBV-LAMAD assay is a rapid, simplified, sensitive, reliable, and easy-to-use detection protocol that can be used as a competitive potential diagnostic/screening tool for EBV infection in clinical settings, especially in basic laboratories in resource-limited regions.

## Data Availability

The datasets generated during and/or analyzed during the current study are available from the corresponding author on reasonable request.
